# ^18^F-FDG texture analysis predicts the pathological Fuhrman nuclear grade of clear cell renal cell carcinoma

**DOI:** 10.1007/s00261-021-03246-x

**Published:** 2021-08-28

**Authors:** Linhan Zhang, Hongyue Zhao, Huijie Jiang, Hong Zhao, Wei Han, Mengjiao Wang, Peng Fu

**Affiliations:** 1 Department of Nuclear Medicine, The First Affiliated Hospital of Harbin Medical University, Harbin, China; 2 Department of Radiology, The Second Affiliated Hospital of Harbin Medical University, Harbin, China; 3 Department of Nuclear Medicine, ShenZhen People’s Hospital, ShenZhen, China

**Keywords:** Adenocarcinoma, Clear cell, Fuhrman grade, Fluorodeoxyglucose F18, Radiomics

## Abstract

**Purpose:**

This article analyzes the image heterogeneity of clear cell renal cell carcinoma (ccRCC) based on positron emission tomography (PET) and positron emission tomography-computed tomography (PET/CT) texture parameters, and provides a new objective quantitative parameter for predicting pathological Fuhrman nuclear grading before surgery.

**Methods:**

A retrospective analysis was performed on preoperative PET/CT images of 49 patients whose surgical pathology was ccRCC, 27 of whom were low grade (Fuhrman I/II) and 22 of whom were high grade (Fuhrman III/IV). Radiological parameters and standard uptake value (SUV) indicators on PET and computed tomography (CT) images were extracted by using the LIFEx software package. The discriminative ability of each texture parameter was evaluated through receiver operating curve (ROC). Binary logistic regression analysis was used to screen the texture parameters with distinguishing and diagnostic capabilities and whose area under curve (AUC) > 0.5. DeLong's test was used to compare the AUCs of PET texture parameter model and PET/CT texture parameter model with traditional maximum standardized uptake value (SUVmax) model and the ratio of tumor SUVmax to liver SUVmean (SUL)model. In addition, the models with the larger AUCs among the SUV models and texture models were prospectively internally verified.

**Results:**

In the ROC curve analysis, the AUCs of SUVmax model, SUL model, PET texture parameter model, and PET/CT texture parameter model were 0.803, 0.819, 0.873, and 0.926, respectively. The prediction ability of PET texture parameter model or PET/CT texture parameter model was significantly better than SUVmax model (*P* = 0.017, *P* = 0.02), but it was not better than SUL model (*P* = 0.269, *P* = 0.053). In the prospective validation cohort, both the SUL model and the PET/CT texture parameter model had good predictive ability, and the AUCs of them were 0.727 and 0.792, respectively.

**Conclusion:**

PET and PET/CT texture parameter models can improve the prediction ability of ccRCC Fuhrman nuclear grade; SUL model may be the more accurate and easiest way to predict ccRCC Fuhrman nuclear grade.

**Graphic abstract:**

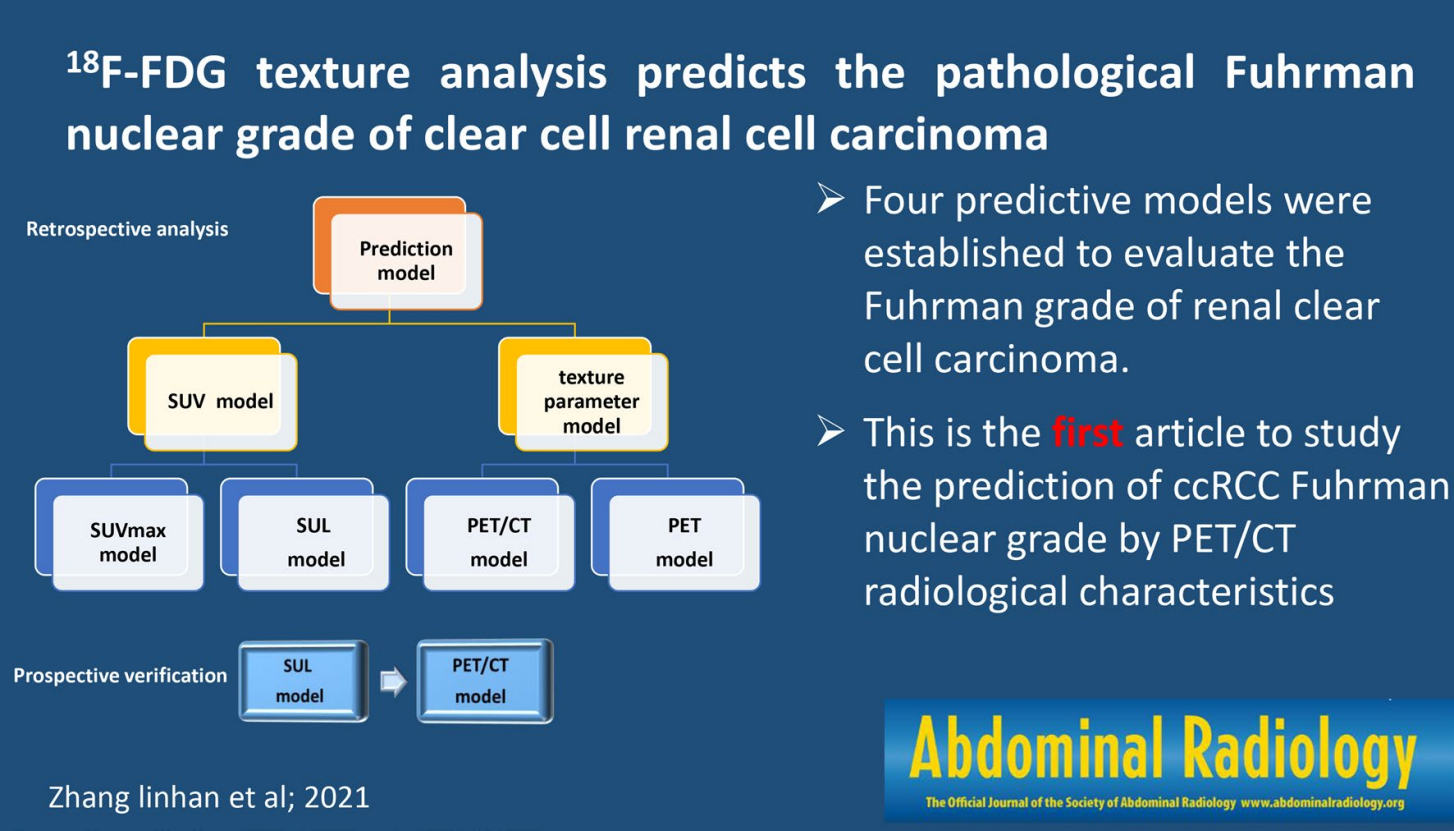

**Supplementary Information:**

The online version contains supplementary material available at 10.1007/s00261-021-03246-x.

## Introduction

According to the cases announced by the American Cancer Society in 2021, the number of new kidney cancer cases was 76,080, and the number of new kidney cancer deaths was 13,780 [1]. Renal cell carcinoma accounts for about 80% of all kidney cancers. Saad et al. [2] conducted an epidemiological study of renal cell carcinoma in the USA in the past 20 years. They found that the incidence of renal cell carcinoma remained stable since 2008 and the overall mortality rate began to decline since 2001. However, as the most common subtype of renal cell carcinoma, clear cell renal cell carcinoma (ccRCC) had a continuously increasing incidence and its mortality rate did not decline until 2012. Therefore, research on ccRCC is of great significance to improve the cure rate of renal tumors. Fuhrman nuclear grade is a recognized prognostic indicator of ccRCC. Studies have shown that [3, 4] low grade ccRCC is associated with a good prognosis and high grade ccRCC is associated with higher infiltration capacity, higher possibility of metastasis, and poor prognosis. In addition, there is a significant difference in the recurrence rate of ccRCC between high grade and low grade patients after surgery, and the risk of recurrence after surgery for higher grade tumors is significantly increased [5]. Therefore, the prediction of ccRCC grade is helpful for clinicians to plan management decisions. In the simplified Fuhrman nuclear grading system, ccRCC cases are divided into low grade (Fuhrman I/II) and high grade (Fuhrman III/IV), which not only reduces the difference between observers, improves repeatability, saves time and money, but also does not affect the ability to predict cancer-specific mortality [6]. Imaging-guided fine-needle aspiration biopsy is the gold standard for preoperative renal tumor grading. However, due to the high spatiotemporal heterogeneity of ccRCC, the biopsy tissue only represents a part of the lesion, which may lead to selection bias and cannot well reflect the Fuhrman nuclear grade of the entire tumor. Moreover, this invasive operation has disadvantages such as poor repeatability and complications. Therefore, non-invasive methods are essential for the preoperative evaluation of ccRCC.

Due to the Warburg effect of malignant tumors, the glucose transporter 1 (GLUT1) is up-regulated, and other enzymes are over-expressed, especially lactate dehydrogenase (LDH), which appear as hypermetabolic foci on ^18^F-fluorodeoxyglucose (^18^F-FDG) positron emission tomography—computed tomography (PET/CT). Therefore, PET/CT is widely used in the diagnosis, grading, monitoring of treatment response, efficacy evaluation, and prognosis determination of various tumors. However, ccRCC does not have the typical Warburg effect, and ^18^F-FDG is excreted through the kidneys. It is difficult to distinguish between tumor metabolism and background. Therefore, in the professional practice guidelines issued by American Urological Association (AUA), and European Society for Medical Oncology (ESMO) and European Association of Urology (EAU), it is generally not recommended to use FDG as a kidney tumor imaging agent [7–9]. But this does not mean that PET/CT examination is useless for the diagnosis of ccRCC. A number of studies have shown that the value of maximum standardized uptake value (SUVmax) as the traditional PET/CT parameter has a certain correlation with the Fuhrman grade of pathology [10–12].

Radiomics that have emerged in recent years can establish models by using a large amount of high-throughput information obtained by image segmentation and feature extraction of regions of interest (ROI) in computed tomography (CT), magnetic resonance imaging (MRI), positron emission tomography (PET), and other images. The researchers input the patient's medical images into computer software with mathematical algorithm functions, and obtain quantitative radiomics characteristics with one-click operation by delineating the target ROI. These features mainly include the shape and geometric characteristics of the lesion, the characteristics of the first-order voxel intensity histogram, the second-order texture features reflecting the spatial arrangement of the voxel intensity, and the high-order features through filters or mathematical transformations. Radiomics can assist physicians to make decisions through deeper mining and analyzing massive image data [13]. Texture analysis is a tool of radiomics through which we can extract texture features from images in a non-invasive manner, which can characterize the histopathological characteristics of living tumors at the molecular level [14]. Although PET parameters based on standard uptake value (SUV) are helpful for the grade of malignant tumors, they cannot reflect the intratumoral heterogeneity (ITH) through the spatial distribution of metabolic activity in the tumor. Texture analysis is one of the most prominent methods to quantify ITH on images. It is an image processing technology that can extract texture information in a quantitative manner and perform mathematical analysis the visually imperceptible changes in pixel intensity. Although texture features are not usually used in the clinical analysis of PET images, there is growing evidence that they have a complementary role in the diagnosis, predicting grade, and treatment of common cancers [15–17]. However, the PET/CT radiomics of kidney tumors is limited by many problems such as the difficulty of segmentation of the ROI and the many factors affecting the feature extraction process. Therefore, we question whether the texture characteristics of PET/CT will significantly improve the predictive ability of the traditional parameters SUV and the ratio of tumor SUVmax to liver SUVmean (SUL) in the grade of ccRCC. Therefore, we established PET texture parameter model, PET/CT texture parameter model, SUVmax model, and SUL model and evaluate the predictive ability of these four models.

## Methods

### Patient selection

A retrospective analysis of the raw image data and basic clinical information of the patients who underwent ^18^F-FDG PET/CT scans and detected kidney tumors in the First Affiliated Hospital of Harbin Medical University from February 2017 to January 2019 was performed. The corresponding prospective internal verification was completed by collecting patient data from February 2019 to December 2019. Inclusion criteria: (1) The surgical pathology was ccRCC; (2) The pathological Fuhrman nuclear grade was certain. Exclusion criteria: (1) Patients who had received any form of treatment before ^18^F-FDG PET/CT scan, including surgery, chemotherapy, radiotherapy, or other methods; (2) The pathological Fuhrman nuclear grade was uncertain, such as patients with Fuhrman nuclear grade II to III; (3) Patients who had obtained pathological results through needle biopsy instead of surgery.

### ^18^F-FDG PET/CT image acquisition and reconstruction

All patients were fasted for 6–8 h, and fasting blood glucose was less than 8 mmol/L, and 3.7–7.4 MBq/kg ^18^F-FDG tracer was injected into the dorsal vein or elbow vein according to the body mass index. The ^18^F-FDG imaging agent was synthesized by the HM-12 cyclotron of Sumitomo Corporation, Japan, and its radiochemical purity was greater than 98%. Images were collected after the patient urinated and rested for 1 h under quiet and dark environment. The whole body ^18^F-FDG PET/CT examination was performed on all patients with Gemini GXL PET/CT scanner (Philips Medical System). Low-dose CT scan was used to attenuation correction for PET image with the following parameters: tube current, 50mAs; tube voltage, 120 kV; slice thickness, 5.0 mm. Then PET scan was performed. PET data were acquired for 1.5 min/bed position, and 6 to 7 bed positions were imaged per patient for whole-body PET scan. Patients do not need to take orally or intravenously inject contrast agent and change their position. According to the institution's standard clinical protocol, the scan range was from the head to the upper thigh. After the PET scan, in order to ensure the quality of the image, standard-dose CT scan was added with the following parameters: tube current, 300mAs; tube voltage, 120 kV. Image registration and the fusion of PET and CT scan images were performed using Syntegra software from Philips, Amsterdam, Netherlands. The images were reconstructed by using line of response (LOR) with 2 mm × 2 mm × 2 mm voxels, and corrections for scatter and random coincidences, while post-reconstruction filtering was not required.

### Radiomic texture analysis

Texture parameters and SUV indicators were extracted using the LifeX software package (version 6.00, http://www.lifexsoft.org) on PET and CT images. The default parameter values of the software package were used. For PET and CT images, spatial resampling parameter was 4.0 mm × 4.0 mm × 4.0 mm and 1.169921875 mm × 1.169921875 mm × 2.5 mm, intensity discretization was 64.0 bin and 400.0 bin, intensity rescaling was 0.0 ~ 20.0 and − 1000.0 ~ 3000.0HU, respectively.

**Fig.1:**
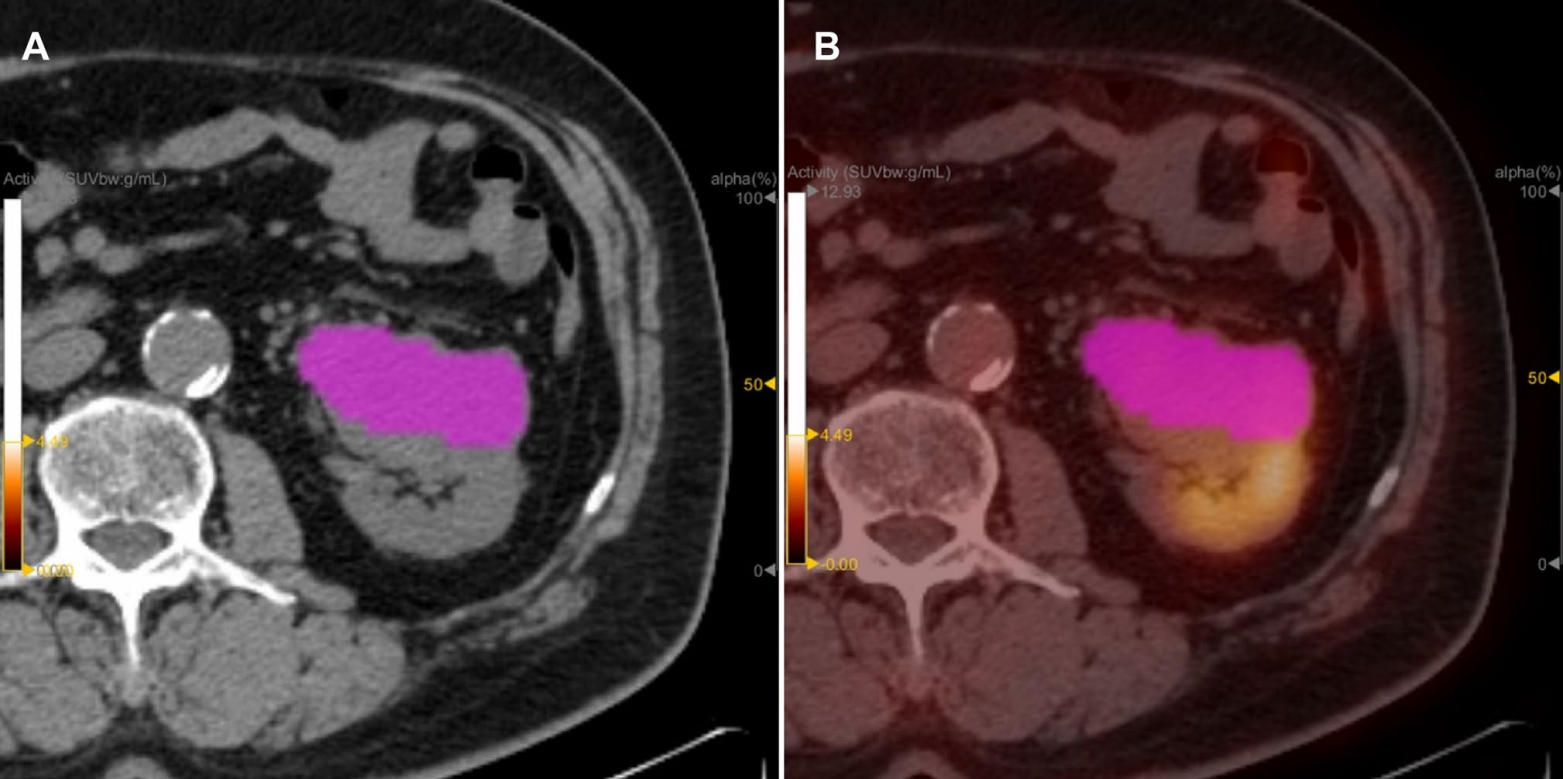
An example of artificially drawing a ROI for clear cell renal cancer in the same patient was shown. The ROI was drawn on the CT image (**A**), then mapped it to the PET image on the same machine to get the ROI of the PET/CT (**B**). The ROI included the cystic part and the necrotic part of the tumor

Tumor segmentation: The ROI was drawn on the image of the corresponding standard-dose CT scan, which included the part of cystic degeneration and necrosis (Fig. 1) and excluded the interference of the physiological ^18^F-FDG remaining in the adjacent renal pelvis and ureter on the fusion image. Without knowing the final histopathological results, two experienced radiology and nuclear medicine associate chief physicians worked together to draw the ROI. If they have a disagreement, another experienced chief physician will attend, and they will discuss and determine. If the ROI does not reach the minimum number of 64 voxels, the case was excluded.

Tumor texture extraction: SUV indicators and texture features were extracted on PET and CT images using LifeX software package. SUV indicators of tumor tissue include minimum standardized uptake value (SUVmin), mean standardized uptake value (SUVmean), maximum standardized uptake value (SUVmax), and total lesion glycolysis (TLG). All texture parameters were divided into six groups: histogram (HISTO), shape (SHAPE), gray-level co-occurrence matrix (GLCM), gray-level run-length matrix (GLRLM), neighborhood gray-level difference matrix (NGLDM), and gray-level region Length matrix (GLZLM). A total of 42 texture parameters were found, including (1) Five histogram features: Skewness, Kurtosis, Entropy_log10, Entropy_log2, and Energy. (2) Five shape features: Volume (ml), Volume (vx), Sphericity, Surface (mm^2^), and Compacity. (3) Seven GLCM functions: Homogeneity, Energy, Contrast, Correlation, Entropy_log10, Entropy_log2, and Dissimilarity. (4) Eleven GLRLM functions: Short-run emphasis (SRE), Long-run emphasis (LRE), Low gray-level run emphasis (LGRE), High gray-level run emphasis (HGRE),Short-run low gray-level emphasis (SRLGE), Short-run high gray-level emphasis (SRHGE), Long-run low gray-level emphasis(LRLGE), Long-run high gray-level emphasis (LRHGE), Gray-level non-uniformity for run (GLNU), Run length non-uniformity (RLNU), and Run percentage (RP). (5) Three NGLDM features: Coarseness, Contrast, and Busyness. (6) Eleven GLZLM features: Short-zone emphasis (SZE), Long-zone emphasis (LZE), Low gray-level zone emphasis (LGZE), High gray-level zone emphasis (HGZE), Short-zone low gray-level emphasis (SZLGE), Short-zone high gray-level emphasis (SZHGE), Long-zone low gray-level emphasis (LZLGE), Long-zone high gray-level emphasis (LZHGE), Gray-level non-uniformity for zone (GLNU), Zone length non-uniformity (ZLNU), and Zone percentage (ZP).

Delineate normal liver tissue and record the SUVmean of healthy liver tissue: Normal liver tissue was delineated in the software and the SUVmean was recorded, while liver lesions and larger blood vessels in the liver were avoided. SUL was used as the evaluation index.

### Histopathological analysis

All tumors were surgically removed to obtain tissue samples, and their ccRCC Fuhrman nuclear grade were obtained through pathological results. Cases of grade III and IV were considered high grade tumors, and cases of grade I and II were considered low grade tumors.

### Statistical analysis

Statistical analysis was performed using SPSS (v25.0, IBM, USA) and MedCalc (v19.4.1, Ostend, Belgium) software. The normality of each parameter was checked using Shapiro–Wilk test. The measurement data that conformed to the normal distribution were expressed as mean ± standard deviation*,* and those that did not conform to the normal distribution were expressed as median (P25, P75). All metabolic data and texture data were divided into low grade and high grade tumor by receiver operating curve (ROC), and the corresponding area under the ROC curve (AUC) was calculated. The texture parameter with distinguishing and diagnostic capabilities was selected when its AUC value was higher than 0.5. The logistic regression analysis was used to evaluate the relationship between the ccRCC Fuhrman nuclear grade and the selected texture parameters. Model variable selection was based on stepwise criteria. The final models were used to generate the ROC. The AUC, sensitivity, specificity, negative predictive value (NPV), and positive predictive value (PPV) were compared. The AUCs were compared using DeLong's test [18]. In addition, the baseline information of patients was compared using T-test, Chi-square test, and Fisher’s exact test.

## Result

### Baseline characteristics

In the retrospective analysis cohort, of a total of 93 patients, 49 patients met the criteria, and there were 27 cases of low grade ccRCC (5 cases of Fuhrman grade I, 22 cases of grade II) and 22 cases of high grade ccRCC (16 cases of Fuhrman grade III, 6 cases of grade IV). The age ranged from 37 to 82 (60.06 ± 1.66) years old, and there was no statistical difference in age in the ccRCC Fuhrman nuclear grade (*t* = 0.269, *P* = 0.607). There were 29 males and 20 females. There was no statistical difference in the grading of ccRCC by gender (*χ*^2^ = 3.032, *P* = 0.082).

In the prospective validation cohort, of a total of 53 patients, 25 patients met the criteria, and there were 14 cases of low grade ccRCC (2 cases of Fuhrman grade I, 12 cases of grade II) and 11 cases of high grade ccRCC (6 cases of Fuhrman grade III, 5 cases of grade IV). The age ranged from 41 to 79 (64.80 ± 0.35) years old, and there was no statistical difference in age in the ccRCC Fuhrman nuclear grade (*t* = 0.069, *P* = 0.946). There were 16 males and 9 females. There was no statistical difference in the grading of ccRCC by gender (*P* = 0.677) (Table 1).

**Table 1:** Basic information of the retrospective analysis cohort and the prospective validation cohort

	Retrospective analysis cohort			Prospective validation cohort		
	Low grade	High grade	*P* value	Low grade	High grade	*P* value
Sex			0.082			0.677
Male	13(48.1%)	16(72.7%)		8(57.1%)	8(72.7%)	
Female	14(51.9%)	6(27.3%)		6(42.9%)	3(27.3%)	
Age	59.63 ± 2.39	60.59 ± 2.39	0.607	64.93 ± 3.19	64.64 ± 2.56	0.946

### Differences of conventional PET parameters in the Fuhrman grades of ccRCC

Except for SUVmin, the conventional PET parameters were statistically different in predicting ccRCC Fuhrman nuclear grade (Table 2). The ability to predict ccRCC Fuhrman nuclear grade according to the AUCs of routine parameters were ranked as: SUL > SUVmax > SUVmean > TLG.

**Table 2:** Differences of conventional PET parameters in the Fuhrmangrades of ccRCC

Conventional parameters	Low grade (*N* = 27)	High grade (*N* = 22)	AUC	SEM	*P*	95%CI
SUVmin	0.75 (0.53–1.02)	0.96 (0.51–1.35)	0.594	0.086	0.26	0.425–0.763
SUVmean	1.75 (1.41–2.15)	2.36 (1.94–2.95)	0.779	0.067	0.001	0.648–0.911
SUVmax	3.42 (2.59–3.74)	4.72 (3.56–5.72)	0.803	0.065	< 0.001	0.677–0.93
TLG(mL)	91.32 (27.81–123.15)	248.38 (28.78–589.76)	0.685	0.081	0.027	0.525–0.845
SUL	1.39 (1.22–1.63)	2.13 (1.56–2.91)	0.818	0.061	< 0.001	0.698–0.938

### Radiomic parameters

The ability of a single texture parameter to predict the ccRCC Fuhrman nuclear grade was shown (Table 3). Among the 42 PET texture features, there were 2 in HISTO features, 4 in GLCM features, 3 in GLRLM features, and 4 in GLZLM features that had good discriminative ability and diagnostic performance. However, SHAPE features and NGLDM features were limited in distinguishing clear cell carcinoma grade. Among CT texture features, 1 in GLRLM feature and 1 in GLZLM feature had good discriminating ability.

**Table 3:** The ability of texture features to distinguish the grade of clear cell carcinoma

Texture parameter	Low grade (*N* = 27)	High grade (*N* = 22)	AUC	SEM	*P*	95%CI
HISTO_Entropy_log10(PET)	0.77 (0.69–0.79)	0.89 (0.76–0.99)	0.746	0.073	0.003	0.602–0.89
HISTO_Entropy_log2(PET)	2.57 (2.29–2.64)	2.96 (2.52–3.28)	0.746	0.073	0.003	0.602–0.89
GLCM_Contrast(PET)	1.53 (1.24–1.8)	2.39 (1.41–3.92)	0.746	0.073	0.003	0.603–0.888
GLCM_Entropy_log10(PET)	1.38 (1.19–1.43)	1.59 (1.37–1.79)	0.746	0.072	0.003	0.604–0.887
GLCM_Entropy_log2(PET)	4.57 (3.96–4.76)	5.27 (4.54–5.93)	0.746	0.072	0.003	0.604–0.887
GLCM_Dissimilarity(PET)	0.9 (0.79–1)	1.15 (0.87–1.51)	0.747	0.072	0.003	0.607–0.888
GLRLM_HGRE(PET)	41.15 (28.19–56.18)	67.96 (51.61–106.44)	0.790	0.066	0.001	0.661–0.918
GLRLM_SRHGE(PET)	31.48 (23.26–46.33)	57.08 (41.82–92.72)	0.786	0.066	0.001	0.656–0.916
GLRLM_LRHGE(PET)	106.89 (67.99–129.83)	151.38 (104.74–195.83)	0.739	0.072	0.004	0.598–0.88
GLZLM_HGZE(PET)	52.9 (39.2–64.27)	92.44 (60.56–124.45)	0.811	0.062	0.000	0.69–0.933
GLZLM_SZHGE(PET)	20.04 (14.38–24.74)	37.58 (24.86–60.17)	0.768	0.075	0.001	0.621–0.915
GLZLM_GLNU(PET)	3.74 (2.65–7.37)	9.47 (2.26–13.88)	0.666	0.086	0.048	0.497–0.835
GLZLM_ZLNU(PET)	4.88 (2.4–6.35)	14.56 (5.08–36.88)	0.719	0.081	0.009	0.561–0.877
GLRLM_HGRE(CT)	10,548.84 (10,501.66–10,634.58)	10,737.51 (10,661.28–10,794.27)	0.879	0.047	0.000	0.787–0.971
GLZLM_HGZE(CT)	10,275.65 (10,103.23–10,413.19337)	10,504.91 (10,361.19–10,566.41)	0.857	0.052	0.000	0.756–0.958

### PET texture parameter model

Regression equation:

$${\text{PREPET}} = 0.058\;{\text{GLRLM}}\_{\text{HGRE}} + 0.197\;{\text{GLZLM}}\_{\text{GLNU}} - 4.978.$$

### PET/CT texture parameter model

Regression equation:

$${\text{PREPET/CT}} = 0.046\;{\text{GLRLM}}\_{\text{HGRE}}\;({\text{PET}}) + 0.01\;{\text{GLZLM}}\_{\text{HGZE}}\;({\text{CT}}) - 193.379.$$

**Fig. 2:**
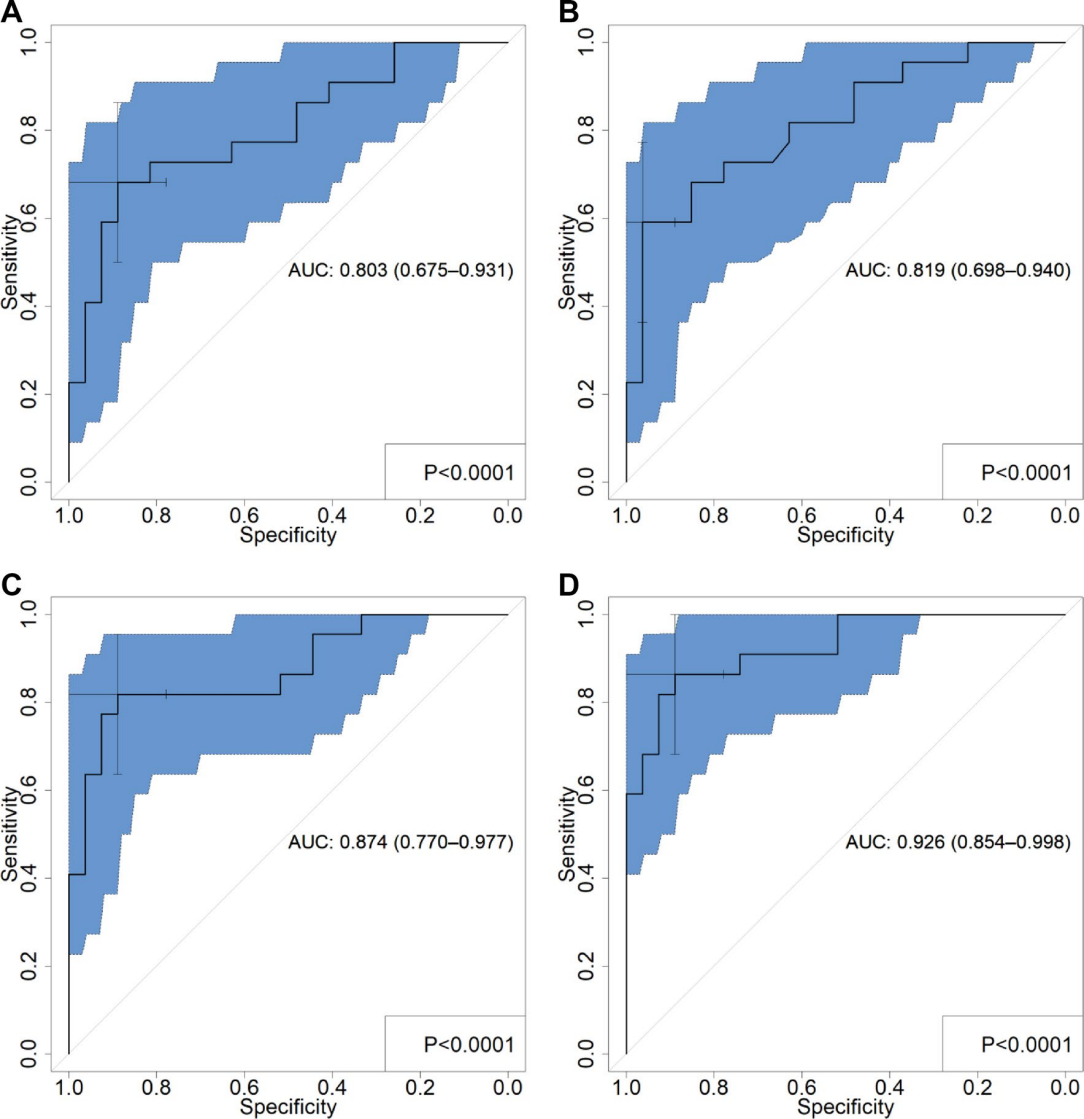
The ROC graphs of SUV model and texture parameter models in predicting ccRCC Fuhrman nuclear grade was shown (**A** SUVmax model; **B** SUL model; **C** PET texture parameter model; **D** PET/CT texture parameter model). The blue area represents the 95% confidence interval, and the cross-marked point represents the best threshold point

Comparison of SUV model and texture parameter model in predicting the ability to ccRCC Fuhrman nuclear grade was shown (Table 4; Fig. 2). The SUVmax model predicted ccRCC Fuhrman nuclear grade with a sensitivity of 68.18%, a specificity of 88.89%, a PPV of 71.4%, and a NPV of 75%. The SUL model had a sensitivity of 59.09%, a specificity of 96.3%, a PPV of 92.9%, and a NPV of 74.3%. SUVmax model and SUL model had no statistical difference in predicting the AUCs of ccRCC Fuhrman nuclear grade (*P* = 0.725) (Table 5). Compared with the SUV model, the PET texture parameter model and the PET/CT texture parameter model both showed better predictive ability, sensitivity (81.82% and 86.36%), specificity (88.89% and 88.89%), and PPV (88.2% and 86.4%), NPV (78.1% and 88.9%), but there was no statistical difference in AUCs between the two texture parameter models (*P* = 0.171). The AUCs of the texture parameter models established by the logistic regression method were greater than that of the SUV models. The difference in AUCs was statistically significant between the PET texture parameter model and the SUVmax model, and between the PET/CT texture parameter model and the SUVmax model (*P* = 0.017, 0.02). However, there was no statistically significant difference in AUCs between the PET texture parameter model and the SUL model, and between the PET/CT texture parameter model and the SUL model (*P* = 0.269, 0.053).

**Table 4:** Comparison of the difference in predictive ability between the texture parameter models and the SUV models

Model	Cut-off	Sensitivity (%)	Specificity (%)	PPV (%)	NPV (%)	AUC	*P* value
SUVmax model	> 4.11	68.18	88.89	71.4	75	0.803	< 0.0001
SUL model	> 1.91	59.09	96.3	92.9	74.3	0.819	< 0.0001
PET texture parameter model	> − 0.45	81.82	88.89	88.2	78.1	0.873	< 0.0001
PET/CT texture parameter model	> − 87.1	86.36	88.89	86.4	88.9	0.926	< 0.0001

*P* refers to the significance for ROC curves

**Table 5:** DeLong test within different models

Model	*P* value
SUVmax model VS SUL model	0.725
PET texture parameter model VS PET/CT texture parameter model	0.171
PET/CT texture parameter model VS SUL model	0.0529
SUVmax model VS PET/CT texture parameter model	0.02
SUL model VS PET texture parameter model	0.2691
SUVmax model VS PET texture parameter model	0.017

### Prospective verification

**Fig. 3:**
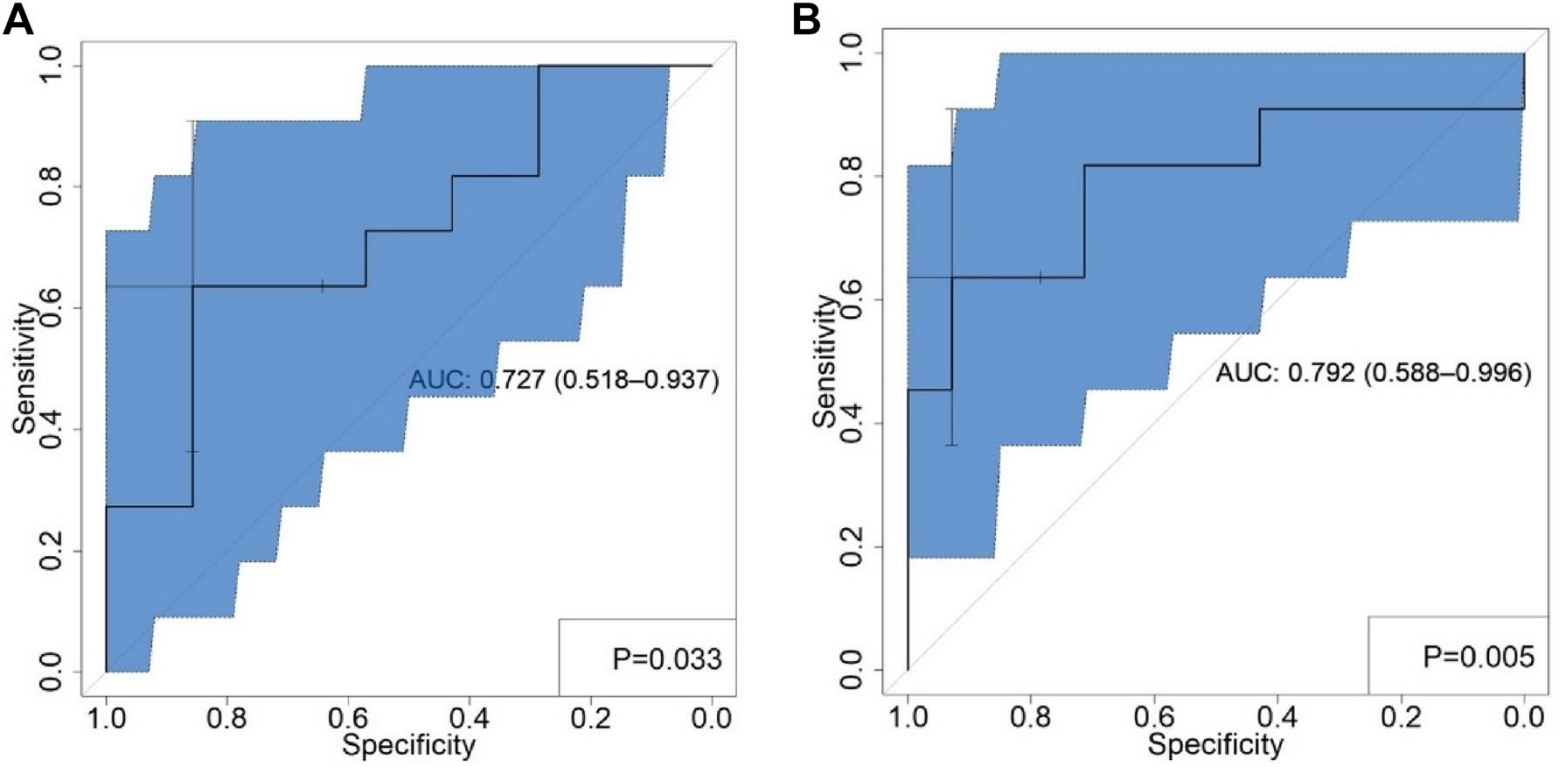
The ROC graphs of SUL model and PET/CT texture parameter model in predicting ccRCC Fuhrman nuclear grade in the prospective validation cohort was shown (**A** SUL model; **B** PET/CT texture parameter model). The blue area represents the 95% confidence interval, and the cross-marked point represents the best threshold point

Using the regression equation in the retrospective analysis cohort, we selected one model with larger AUCs of SUV models and texture parameter models, respectively and prospectively verified their ability to predict the Fuhrman grade of ccRCC in 25 patients. The AUCs of SUL model and PET/CT texture parameter model were 0.727 and 0.792, respectively. Although the predictive abilities of the SUL model and PET/CT texture parameter model are lower than in retrospective analysis cohort, they still have better predictive abilities (Table 6; Fig. 3), and there was no statistically significant difference in AUCs between the two models (*P* = 0.489).

**Table 6:** Predictive ability of SUL model and PET/CT texture parameter model in the prospective validation cohort

Model	Sensitivity (%)	Specificity (%)	PPV (%)	NPV (%)	AUC	*P* value
SUL model	63.64	85.71	77.8	75	0.727	0.033
PET/CT texture parameter model	63.64	92.86	87.5	76.5	0.792	0.0049

*P* refers to the significance for ROC curves

## Discussion

The results of our study indicate that ^18^F-FDG PET/CT SUV indicators and radiological parameters can assist in predicting ccRCC Fuhrman nuclear grade. Among the four discriminant models in the retrospective analysis cohort, the PET/CT texture parameter model had the highest sensitivity and NPV, and higher specificity and PPV. The SUL model had the highest specificity, the lowest sensitivity, the highest PPV, and the lowest NPV. PET and PET/CT texture parameter models (AUC = 0.874, 0.926) had more predictive ability than SUVmax model (AUC = 0.803); Compared with the SUL model (AUC = 0.819), PET and PET/CT texture parameter models (AUC = 0.874, 0.926) could improve the predictive ability, but it was not statistically significant (*P* = 0.269, 0.053). In the prospective validation cohort, the predictive ability of the SUL model and the PET/CT texture parameter model decreased (AUC = 0.727, 0.792), but both models showed the ability to distinguish the high and low Fuhrman nuclear grade of ccRCC, and there was no statistical difference in the AUCs of the two models.

As far as we know, this is the first article to study the prediction of ccRCC Fuhrman nuclear grade by PET/CT radiomics characteristics. In previous studies on ^18^F-FDG PET/CT to predict ccRCC Fuhrman nuclear grade, SUVmax was the most commonly used index [10, 19]. SUVmax can be affected by many factors, such as blood sugar level, temperature, muscle activity, renal background metabolism, etc. There are still many shortcomings in the grading of kidney cancer based on SUVmax alone, so the standardization of SUVmax may be more meaningful to distinguish between high and low grade of ccRCC. Since the uptake of ^18^F-FDG in the liver blood pool is relatively stable, it is often used as a reference standard in some studies [20]. Nado et al. [21] did some researches on the correlation between tumor SUVmax, tumor SUL, and pathological Fuhrman nuclear grade, and they found that the AUC of tumor SUL was greater than that of tumor SUVmax, but there was no significant difference, which is consistent with our results. In terms of ROI drawing, since many tumors only show low level uptake of ^18^F-FDG, it is impossible to segment the tumor area directly from the PET image through freehand or semi-automated methods such as percentage threshold or fuzzy local adaptive Bayesian methods. Therefore, ROIs were drawn on the corresponding CT image in our research, which covered almost the tumor tissue and the entire healthy liver tissue excluding the lesions and blood vessels in the liver. Compared with the research of Nado [21], our research is more objective, comprehensive and accurate in reflecting the metabolism of tumor and liver tissue. The ROIs drawn by different observers may be different, which will affect the analysis of texture features. Based on the image characteristics of ccRCC, in order to reduce the difference between observers, two physicians were engaged in drawing the ROI in our study. When they had a disagreement, a third physician participated, and the three physicians negotiated and determined together. Current researches mainly focus on the relationship between CT, MR texture analysis, and ccRCC Fuhrman nuclear grade [22–27]. Since PET texture analysis can better reflect the heterogeneity of tumors than conventional radiomics, the emergence of PET radiomics provides new possibilities for the prediction of ccRCC Fuhrman nuclear grade. Although the ^18^F-FDG PET image does not have high spatial resolution, it may better reflect the situation of the tumor than the traditional parameters or morphological characteristics, which quantified the uptake distribution within the tumor through radiomics feature, and this has been verified in many tumors [28–30]. At present, there are few articles on studying the PET radiomics of kidney cancer. Wang et al. [31] found that PET texture parameters can predict the overall survival of patients with renal lymphoma or adrenal lymphoma. ZHU et al. [32] used PET texture analysis method to distinguish renal cell carcinoma and renal lymphoma. They created a new variable through binary logistic regression analysis of various parameters of HISTO, SHAPE, PARAMS, GLCM, GLRLM, NGLDM, and GLZLM, and determined the AUC, cutoff value, sensitivity, and specificity of this new variable. However, this study cannot eliminate the collinearity between various indicators. Our research selected the single variable with the strongest diagnostic ability and distinguishing grading ability among texture indicators, and screened the statistically significant parameters for predicting grading by stepwise regression, which can eliminate the collinearity between various parameters and avoid overfitting of the regression equation.

Our research also has certain limitations. The main limitation is the relatively small sample size. On the one hand, fewer patients come to our center due to the limited value of ^18^F-FDG PET/CT in the qualitative diagnosis of renal tumors. Most of the confirmed patients come to check for metastasis or recurrence, while they have lost the opportunity for surgery. On the other hand, PET radiomics features are very sensitive to changes in acquisition methods, image reconstruction algorithms, number of iterations or subsets, and acquisition time after injection [33–35], which largely limits the development of multi-center studies. Although there are currently some methods to eliminate the influence of multiple sites and different scanners on texture features [36, 37], such as using conditional generative adversarial networks (cGANs) or ComBat, they are still in the research stage and there is no authoritative standardized guideline. Therefore, the sample size of patients who meet the research conditions is small. With the popularization of PET radiomics, it is necessary to conduct further research and verification on larger samples in the future to confirm our findings.

## Conclusion

Although this study has some shortcomings, it can still be proved that ^18^F-FDG PET/CT radiomic parameters have a significant positive effect on the pathological Fuhrman nuclear grade of ccRCC, and will have a greater effect in improving the diagnosis and treatment of renal cancer. At the same time, the tumor SUL is also a promising method because of its simple operation and high ability to predict the Fuhrman nuclear grade of ccRCC pathology.

## Supplementary Information

Below is the link to the electronic supplementary material.

Supplementary file1 (PPTX 600 kb)
