# Supplementary material for: 18F-FDG texture analysis predicts the pathological Fuhrman nuclear grade of clear cell renal cell carcinoma
Source: Abdom Radiol (NY). 2021 Aug 28;46(12):5618–28. doi: 10.1007/s00261-021-03246-x (PMC8590655; doi:10.1007/s00261-021-03246-x)
Supplement: Supplementary file 1 — Supplementary file1 (PPTX 600 kb) [file 261_2021_3246_MOESM1_ESM.pptx]

## Slide 1
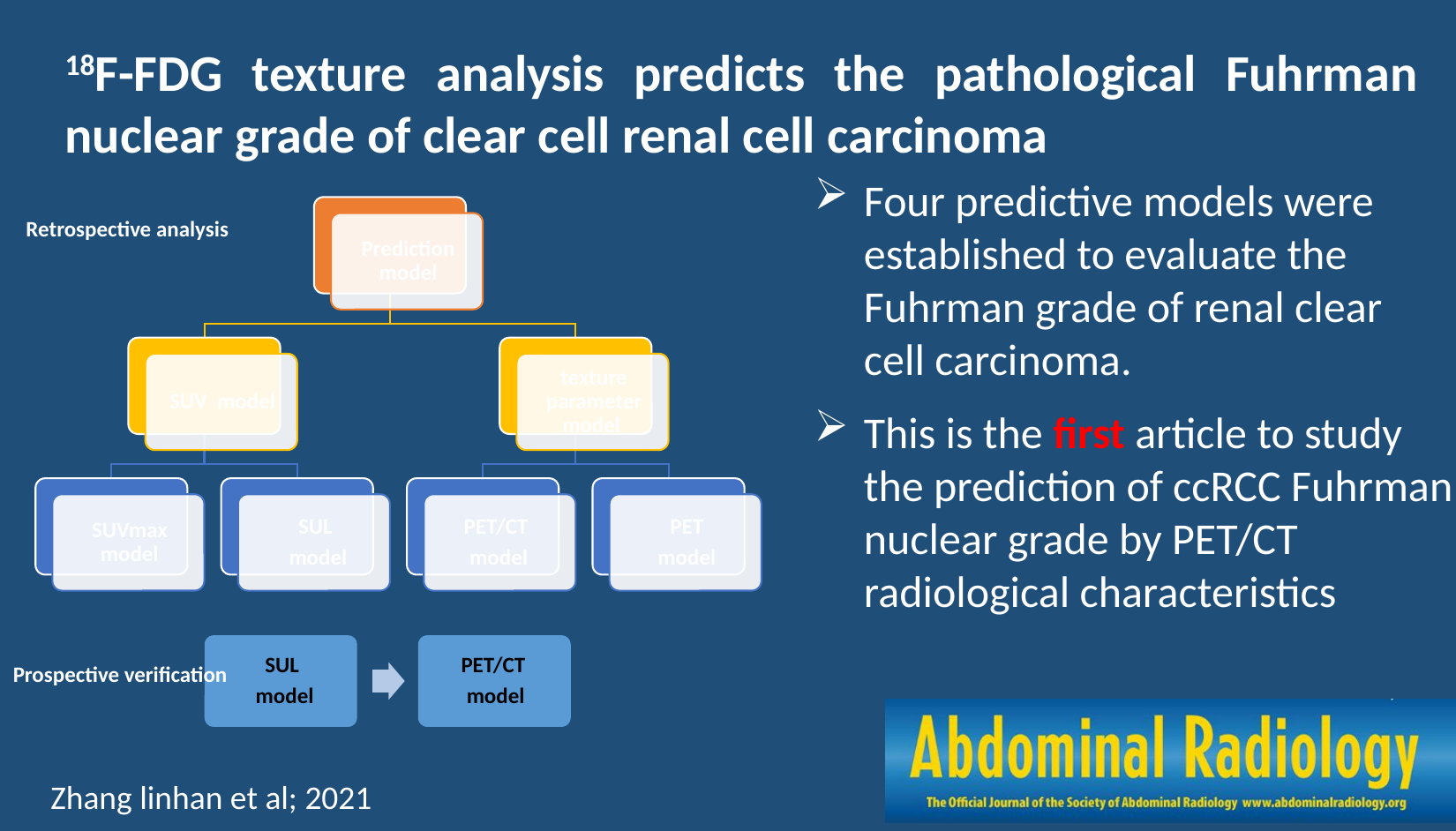

18F-FDG texture analysis predicts the pathological Fuhrman nuclear grade of clear cell renal cell carcinoma
Four predictive models were established to evaluate the Fuhrman grade of renal clear cell carcinoma.
Retrospective analysis
This is the first article to study the prediction of ccRCC Fuhrman nuclear grade by PET/CT radiological characteristics
Prospective verification
Zhang linhan et al; 2021

## Slide 2
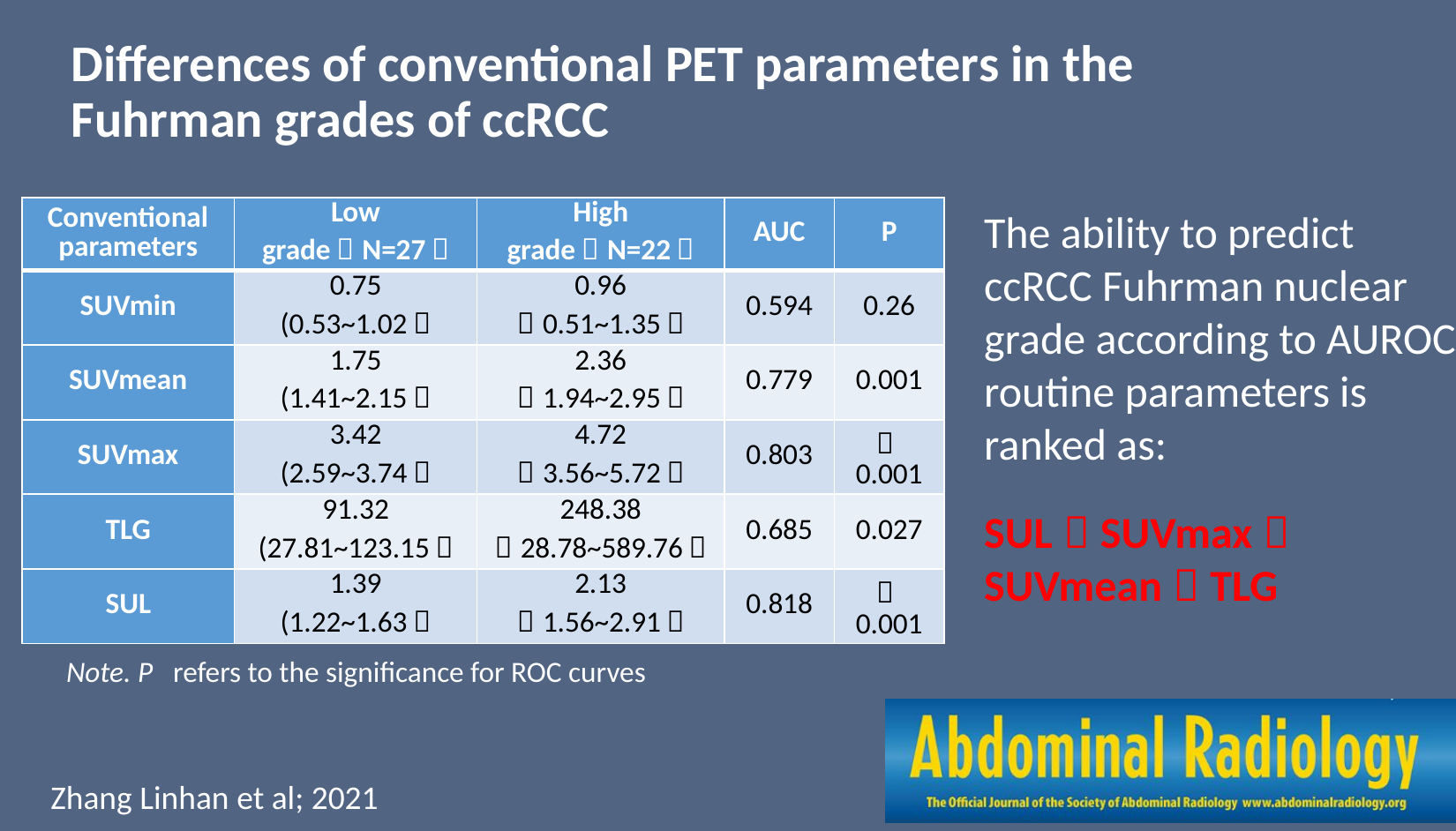

# Differences of conventional PET parameters in the Fuhrman grades of ccRCC
| Conventional parameters | Low grade（N=27） | High grade（N=22） | AUC | P |
| --- | --- | --- | --- | --- |
| SUVmin | 0.75 (0.53~1.02） | 0.96 （0.51~1.35） | 0.594 | 0.26 |
| SUVmean | 1.75 (1.41~2.15） | 2.36 （1.94~2.95） | 0.779 | 0.001 |
| SUVmax | 3.42 (2.59~3.74） | 4.72 （3.56~5.72） | 0.803 | ＜0.001 |
| TLG | 91.32 (27.81~123.15） | 248.38 （28.78~589.76） | 0.685 | 0.027 |
| SUL | 1.39 (1.22~1.63） | 2.13 （1.56~2.91） | 0.818 | ＜0.001 |
The ability to predict ccRCC Fuhrman nuclear grade according to AUROC routine parameters is ranked as:
SUL＞SUVmax＞SUVmean＞TLG
Note. P   refers to the significance for ROC curves
Zhang Linhan et al; 2021

## Slide 3
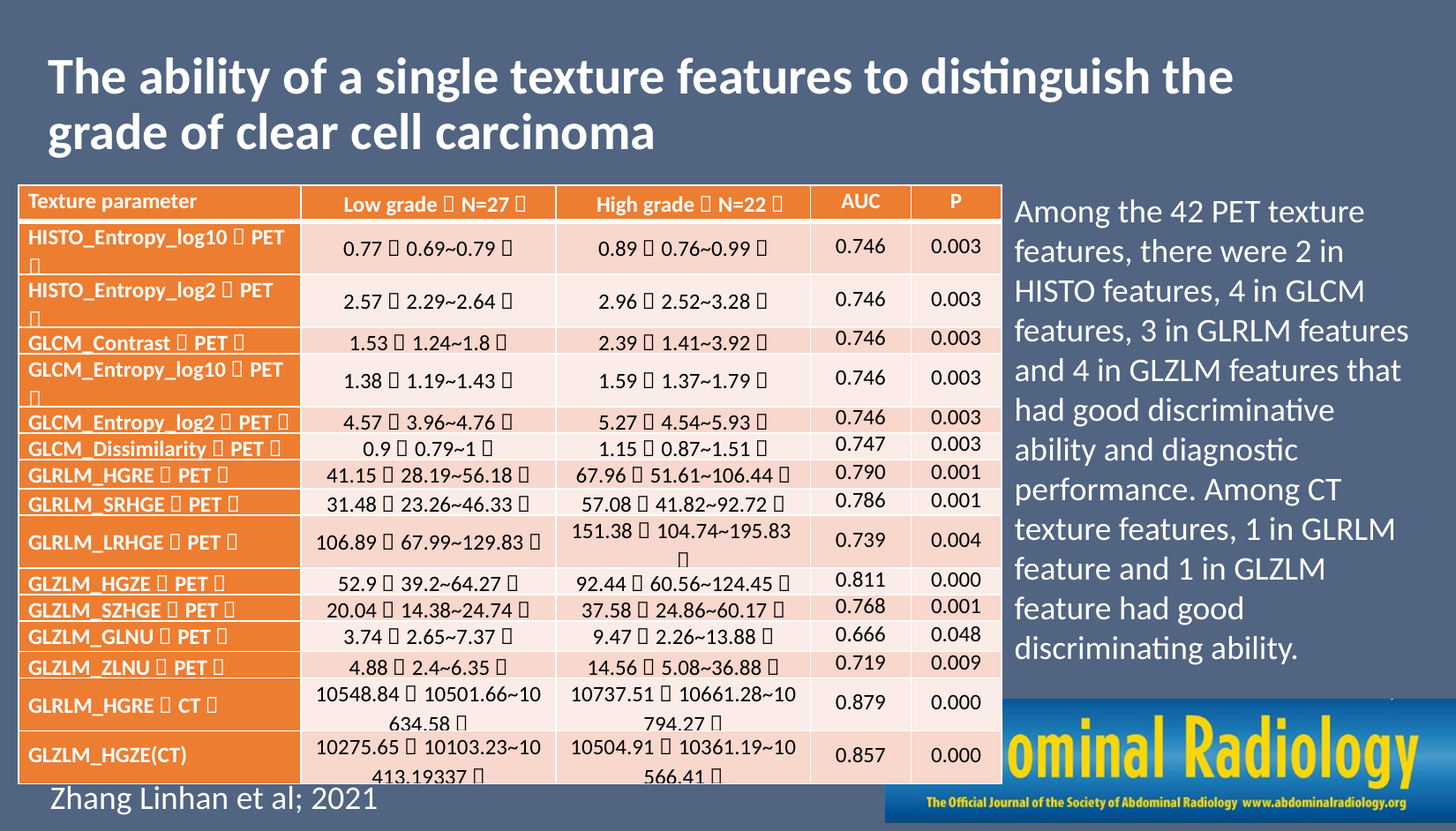

# The ability of a single texture features to distinguish the grade of clear cell carcinoma
Among the 42 PET texture features, there were 2 in HISTO features, 4 in GLCM features, 3 in GLRLM features and 4 in GLZLM features that had good discriminative ability and diagnostic performance. Among CT texture features, 1 in GLRLM feature and 1 in GLZLM feature had good discriminating ability.
| Texture parameter | Low grade（N=27） | High grade（N=22） | AUC | P |
| --- | --- | --- | --- | --- |
| HISTO\_Entropy\_log10（PET） | 0.77（0.69~0.79） | 0.89（0.76~0.99） | 0.746 | 0.003 |
| HISTO\_Entropy\_log2（PET） | 2.57（2.29~2.64） | 2.96（2.52~3.28） | 0.746 | 0.003 |
| GLCM\_Contrast（PET） | 1.53（1.24~1.8） | 2.39（1.41~3.92） | 0.746 | 0.003 |
| GLCM\_Entropy\_log10（PET） | 1.38（1.19~1.43） | 1.59（1.37~1.79） | 0.746 | 0.003 |
| GLCM\_Entropy\_log2（PET） | 4.57（3.96~4.76） | 5.27（4.54~5.93） | 0.746 | 0.003 |
| GLCM\_Dissimilarity（PET） | 0.9（0.79~1） | 1.15（0.87~1.51） | 0.747 | 0.003 |
| GLRLM\_HGRE（PET） | 41.15（28.19~56.18） | 67.96（51.61~106.44） | 0.790 | 0.001 |
| GLRLM\_SRHGE（PET） | 31.48（23.26~46.33） | 57.08（41.82~92.72） | 0.786 | 0.001 |
| GLRLM\_LRHGE（PET） | 106.89（67.99~129.83） | 151.38（104.74~195.83） | 0.739 | 0.004 |
| GLZLM\_HGZE（PET） | 52.9（39.2~64.27） | 92.44（60.56~124.45） | 0.811 | 0.000 |
| GLZLM\_SZHGE（PET） | 20.04（14.38~24.74） | 37.58（24.86~60.17） | 0.768 | 0.001 |
| GLZLM\_GLNU（PET） | 3.74（2.65~7.37） | 9.47（2.26~13.88） | 0.666 | 0.048 |
| GLZLM\_ZLNU（PET） | 4.88（2.4~6.35） | 14.56（5.08~36.88） | 0.719 | 0.009 |
| GLRLM\_HGRE（CT） | 10548.84（10501.66~10634.58） | 10737.51（10661.28~10794.27） | 0.879 | 0.000 |
| GLZLM\_HGZE(CT) | 10275.65（10103.23~10413.19337） | 10504.91（10361.19~10566.41） | 0.857 | 0.000 |
Note. P  refers to the significance for ROC curves.
Zhang Linhan et al; 2021

## Slide 4
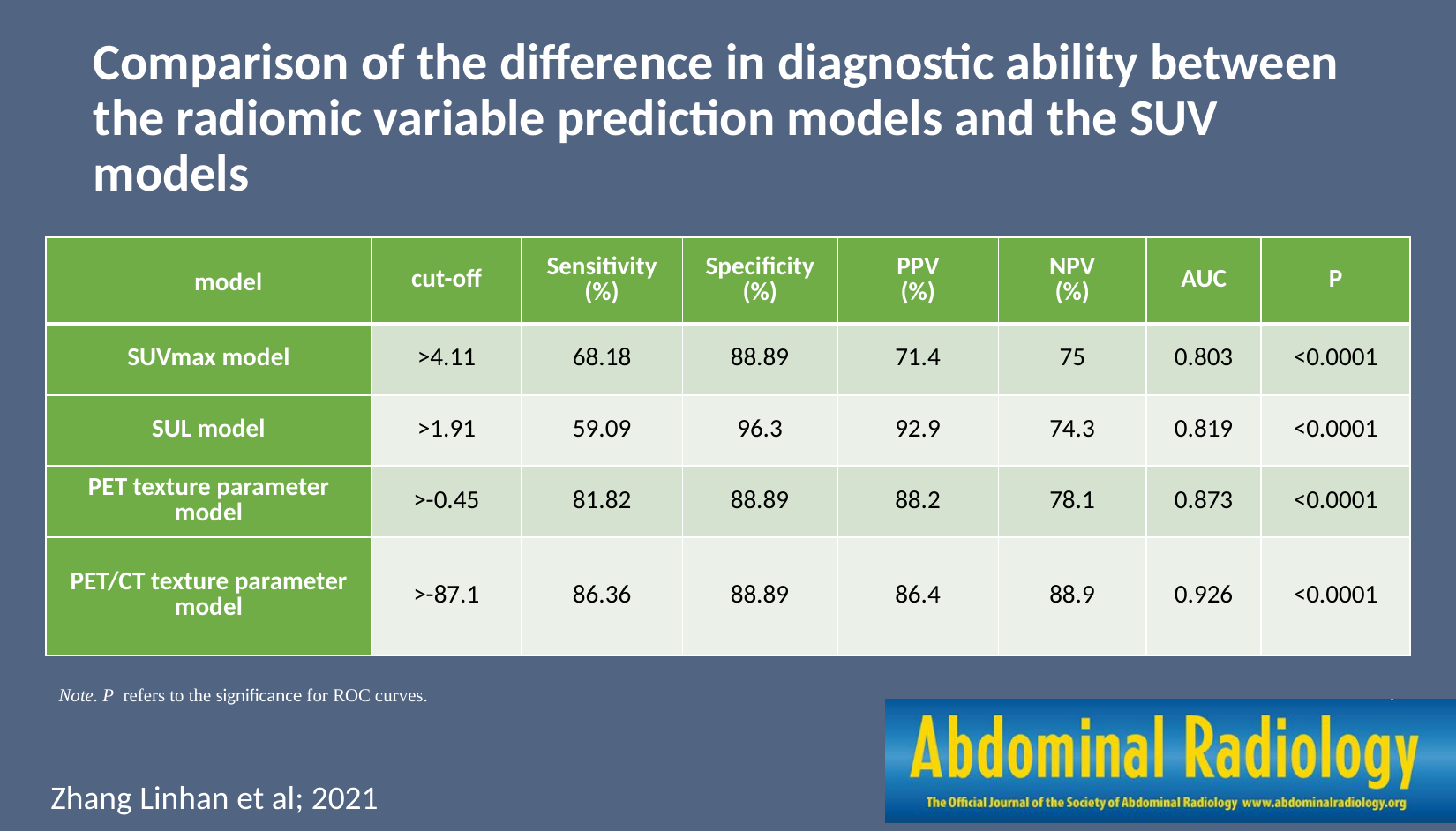

# Comparison of the difference in diagnostic ability between the radiomic variable prediction models and the SUV models
| model | cut-off | Sensitivity (%) | Specificity (%) | PPV (%) | NPV (%) | AUC | P |
| --- | --- | --- | --- | --- | --- | --- | --- |
| SUVmax model | >4.11 | 68.18 | 88.89 | 71.4 | 75 | 0.803 | <0.0001 |
| SUL model | >1.91 | 59.09 | 96.3 | 92.9 | 74.3 | 0.819 | <0.0001 |
| PET texture parameter model | >-0.45 | 81.82 | 88.89 | 88.2 | 78.1 | 0.873 | <0.0001 |
| PET/CT texture parameter model | >-87.1 | 86.36 | 88.89 | 86.4 | 88.9 | 0.926 | <0.0001 |
Note. P  refers to the significance for ROC curves.
Zhang Linhan et al; 2021

## Slide 5
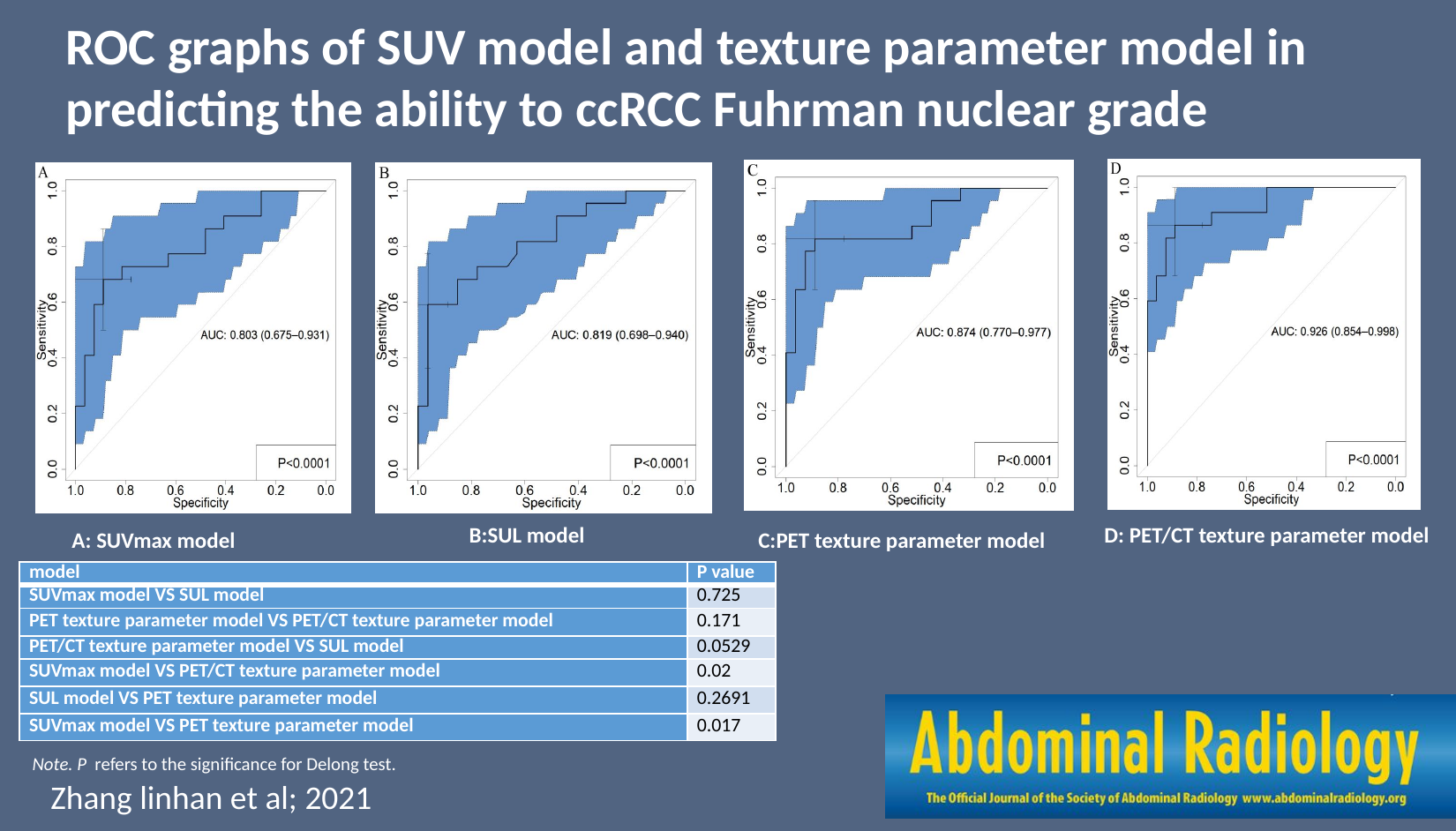

ROC graphs of SUV model and texture parameter model in predicting the ability to ccRCC Fuhrman nuclear grade
B:SUL model
D: PET/CT texture parameter model
A: SUVmax model
C:PET texture parameter model
| model | P value |
| --- | --- |
| SUVmax model VS SUL model | 0.725 |
| PET texture parameter model VS PET/CT texture parameter model | 0.171 |
| PET/CT texture parameter model VS SUL model | 0.0529 |
| SUVmax model VS PET/CT texture parameter model | 0.02 |
| SUL model VS PET texture parameter model | 0.2691 |
| SUVmax model VS PET texture parameter model | 0.017 |
Note. P  refers to the significance for Delong test.
Zhang linhan et al; 2021

## Slide 6
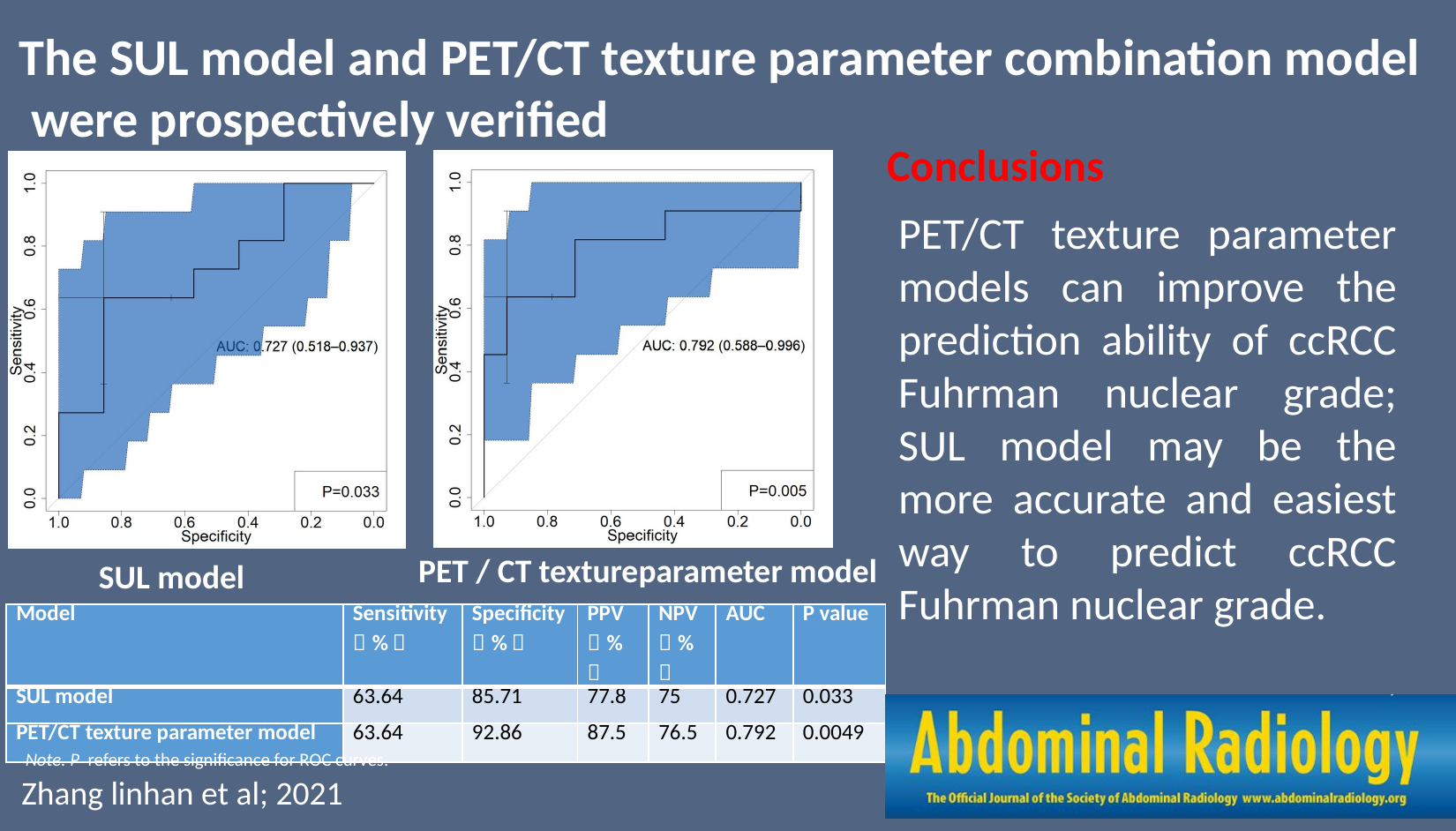

The SUL model and PET/CT texture parameter combination model
 were prospectively verified
Conclusions
PET/CT texture parameter models can improve the prediction ability of ccRCC Fuhrman nuclear grade; SUL model may be the more accurate and easiest way to predict ccRCC Fuhrman nuclear grade.
PET / CT textureparameter model
SUL model
| Model | Sensitivity（%） | Specificity（%） | PPV（%） | NPV（%） | AUC | P value |
| --- | --- | --- | --- | --- | --- | --- |
| SUL model | 63.64 | 85.71 | 77.8 | 75 | 0.727 | 0.033 |
| PET/CT texture parameter model | 63.64 | 92.86 | 87.5 | 76.5 | 0.792 | 0.0049 |
Note. P  refers to the significance for ROC curves.
Zhang linhan et al; 2021
